# Posterior cerebellar Purkinje cells in an SCA5/SPARCA1 mouse model are especially vulnerable to the synergistic effect of loss of β-III spectrin and GLAST

**DOI:** 10.1093/hmg/ddw274

**Published:** 2016-08-15

**Authors:** Emma M. Perkins, Daumante Suminaite, Yvonne L. Clarkson, Sin Kwan Lee, Alastair R. Lyndon, Jeffrey D. Rothstein, David J.A. Wyllie, Kohichi Tanaka, Mandy Jackson

**Affiliations:** 1 The Centre for Integrative Physiology, The University of Edinburgh, Hugh Robson Building, George Square, Edinburgh, UK; 2 School of Energy, Geoscience, Infrastructure and Society, Heriot-Watt University, John Muir Building, Riccarton, Edinburgh, UK; 3 Department of Neurology and Neuroscience, Johns Hopkins University, School of Medicine, Baltimore, MD, USA; 4 Centre for Brain Development and Repair, Institute for Stem Cell Biology and Regenerative Medicine, Bangalore, India; 5 Laboratory of Molecular Neuroscience, Medical Research Institute, Tokyo Medical and Dental University, Bunkyo-Ku, Tokyo, Japan

## Abstract

Clinical phenotypes of spinocerebellar ataxia type-5 (SCA5) and spectrin-associated autosomal recessive cerebellar ataxia type-1 (SPARCA1) are mirrored in mice lacking β-III spectrin (β-III^-/-^). One function of β-III spectrin is the stabilization of the Purkinje cell-specific glutamate transporter EAAT4 at the plasma membrane. In β-III^-/-^ mice EAAT4 levels are reduced from an early age. In contrast levels of the predominant cerebellar glutamate transporter GLAST, expressed in Bergmann glia, only fall progressively from 3 months onwards. Here we elucidated the roles of these two glutamate transporters in cerebellar pathogenesis mediated through loss of β-III spectrin function by studying EAAT4 and GLAST knockout mice as well as crosses of both with β-III^-/-^ mice. Our data demonstrate that EAAT4 loss, but not abnormal AMPA receptor composition, in young β-III^-/-^ mice underlies early Purkinje cell hyper-excitability and that subsequent loss of GLAST, superimposed on the earlier deficiency of EAAT4, is responsible for Purkinje cell loss and progression of motor deficits. Yet the loss of GLAST appears to be independent of EAAT4 loss, highlighting that other aspects of Purkinje cell dysfunction underpin the pathogenic loss of GLAST. Finally, our results demonstrate that Purkinje cells in the posterior cerebellum of β-III^-/-^ mice are most susceptible to the combined loss of EAAT4 and GLAST, with degeneration of proximal dendrites, the site of climbing fibre innervation, most pronounced. This highlights the necessity for efficient glutamate clearance from these regions and identifies dysregulation of glutamatergic neurotransmission particularly within the posterior cerebellum as a key mechanism in SCA5 and SPARCA1 pathogenesis.

## Introduction

Output from the cerebellar cortex sculpts fine control of motor movements and balance and is derived solely from Purkinje cell neurons, alterations to which result in ataxia. Cerebellar abnormalities may also underlie the pathophysiology in Alzheimer’s disease (1,2), schizophrenia (3), autism (4–6) and other cognitive and neuropsychiatric disorders (7–10).

Mutations in the gene encoding β-III spectrin (*SPTBN2*) lead to spinocerebellar ataxia type-5 (SCA5) (11) and spectrin-associated autosomal recessive cerebellar ataxia type-1 (SPARCA1) (12), two human neurodegenerative diseases involving gait ataxia and cerebellar atrophy. β-III spectrin is highly expressed in the cerebellum and the phenotype of β-III^-/-^ mutant mice mirrors the clinical phenotypes of SCA5 and SPARCA1 (12,13). Considerable Purkinje cell dysfunction is detectable in young β-III^-/-^ mutant mice prior to cell loss, including increased parallel fibre-Purkinje cell excitatory postsynaptic currents (PF-PC EPSCs) and a 50% reduction in EAAT4 protein levels (13). A progressive loss of GLAST is also seen from 3 months of age (13). EAAT4 and GLAST are the two principal cerebellar glutamate transporters, and ultrastructural analysis of Purkinje cells from β-III^-/-^ mice revealing dark cell degeneration is consistent with cell death occurring from delayed glutamate-mediated excitotoxicity (13).

We previously demonstrated that β-III spectrin directly interacts with EAAT4 and stabilizes high levels of expression at the plasma membrane (14). The early loss of EAAT4 in β-III^-/-^ mice is, therefore, almost certainly due to loss of the β-III spectrin anchor. However, the cellular and molecular mechanisms responsible for the delayed loss of GLAST in β-III^-/-^ mice are still to be resolved. The present study uses crosses of ET4^-/-^ and GLAST^-/-^ knockout mice with β-III^-/-^ mice to dissect the relative roles of these glutamate transporters in the pathophysiology of motor deficits. We used these genetic approaches to determine the mechanisms underlying initial hyper-excitability in β-III^-/-^ Purkinje cells (13) and identify which factors previously only correlated with ataxia are directly linked to disease, facilitating the development of effective therapeutic strategies.

Here we demonstrate that loss of EAAT4 accounts for the initial hyper-excitability of Purkinje cells lacking β-III spectrin and that loss of GLAST appears to work synergistically to worsen motor deficits. Yet the early loss of EAAT4 does not underlie the subsequent loss of GLAST protein. When levels of both EAAT4 and GLAST are compromised in β-III^-/-^ mice, the proximal dendrites of Purkinje cells within the posterior cerebellum are the most vulnerable to degeneration. This highlights the importance of efficient glutamate clearance in the vicinity of climbing fibre innervation and identifies this region as an important therapeutic target for SCAs.

## Results

### EAAT4 loss results in Purkinje cell hyper-excitability similar to β-III^-/-^ mice

**Figure 1.**
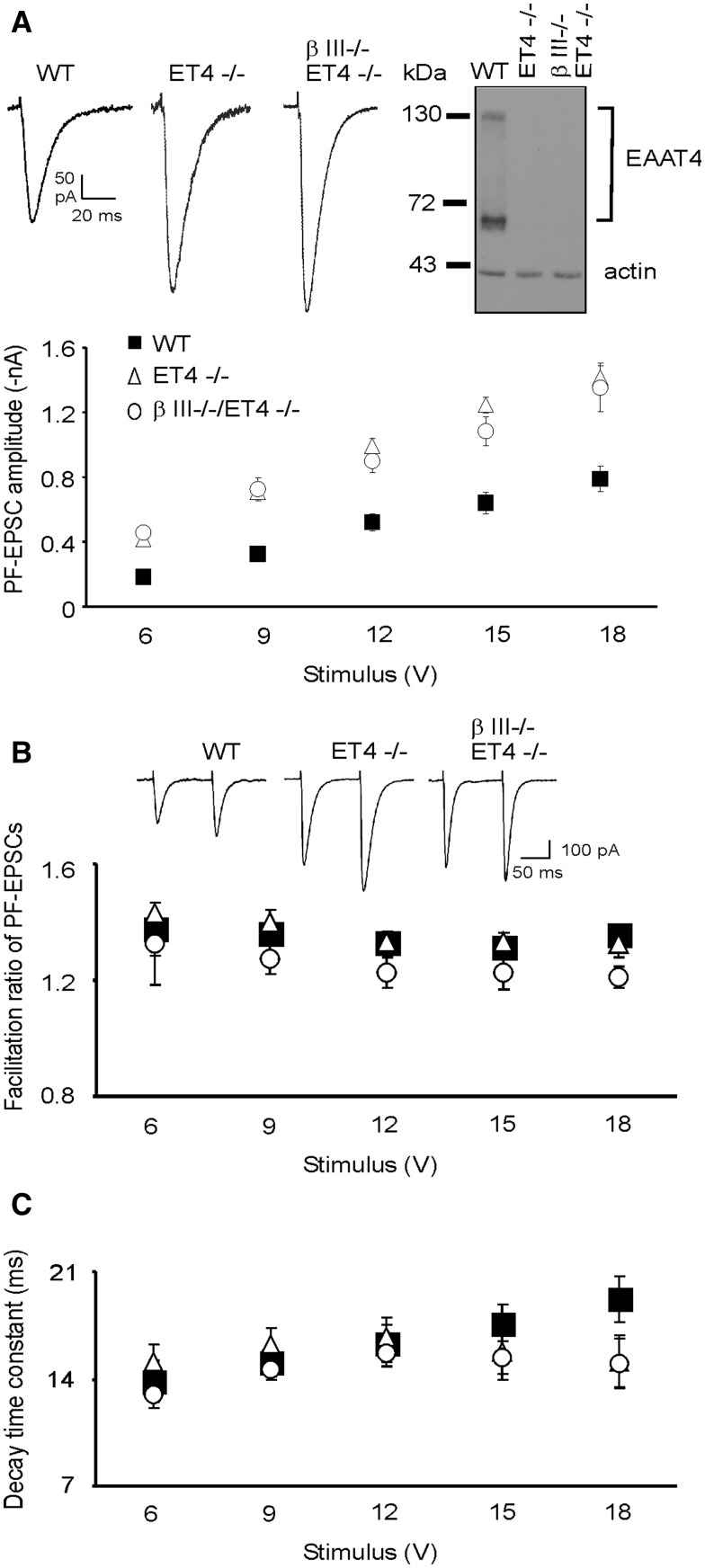
Larger parallel fibre-mediated EPSC amplitude in ET4^-/-^ mice. (**A**) Top, representative EPSC waveforms at 6 V stimulus and immunoblot analysis for all genotypes. Bottom, mean PF-PC EPSC amplitudes versus stimulus intensity for 6-week old WT, ET4^-/-^ and β-III^-/-^/ET4^-/-^ animals. No significant differences detected between ET4^-/-^ and β-III^-/-^/ET4^-/-^ across the range of stimulus intensities but both significantly different to WT animals using mixed model ANOVA analysis. (**B**) Top, examples of PF-EPSC waveforms for pairs of stimuli at 6 V. Bottom, degree of paired-pulse facilitation. (**C**) Single exponential decay time constants. All data are means ± SEM, *N =* 3–5, *n =* 11 (β-III^-/-^/ET4^-/-^), 13 (WT) and 15 (ET4^-/-^).

To discern differences in glutamatergic neurotransmission and investigate the involvement of EAAT4 loss in the previously reported β-III^-/-^ Purkinje cell hyper-excitability (13) we measured PF-mediated EPSC amplitudes at increasing stimulus intensities, as widely reported (15–18). This revealed that PF-EPSCs in 6–week old EAAT4 knockout (ET4^-/-^) mice were significantly larger at all stimulus intensities compared to wild type (WT) (*P =* 1.9 × 10 ^−^ ^5^ Fig. 1A), similar to β-III^-/-^ mice (13). Moreover, there was no significant difference in amplitude between ET4^-/-^ cells and β-III^-/-^/ET4^-/-^ cells (*P =* 0.646) indicating EAAT4 loss underpins the enhanced PF-PC EPSC amplitudes observed in β-III^-/-^ mice (13).

To ascertain whether the increased EPSC amplitudes were a consequence of pre- or post-synaptic effects paired-pulse facilitation was measured by evoking two presynaptic spikes in close succession (Fig. 1B). This provides an indication of the release probability of the pre-synaptic cell by comparing the postsynaptic response of the second spike to the first spike. No difference in the paired-pulse facilitation ratio was seen between 6-week old WT, ET4^-/-^ and β-III^-/-^/ET4^-/-^ mice demonstrating changes in presynaptic release probability from parallel fibre terminals are unlikely to underlie the enhanced EPSC amplitudes in ET4^-/-^ and β-III^-/-^/ET4^-/-^ mice (Fig. 1B). Examination of the decay kinetics revealed the single exponential decay time constant of WT Purkinje cells was similar to the study by Watase et al. (19) for mature Purkinje cells (14.3 ± 3.8 (SD)) and no difference was detected between WT, ET4^-/-^ and β-III^-/-^/ET4^-/-^ animals (Fig. 1C). In contrast the decay kinetics in GLAST^-/-^ mice had a single exponential decay time constant 36% longer than WT mice (*P =* 0.018; data not shown). This is in agreement with other studies (20,21), and further validates our PF-EPSC data.

### No difference in AMPA receptor composition in β-III^-/-^mice

**Figure 2.**
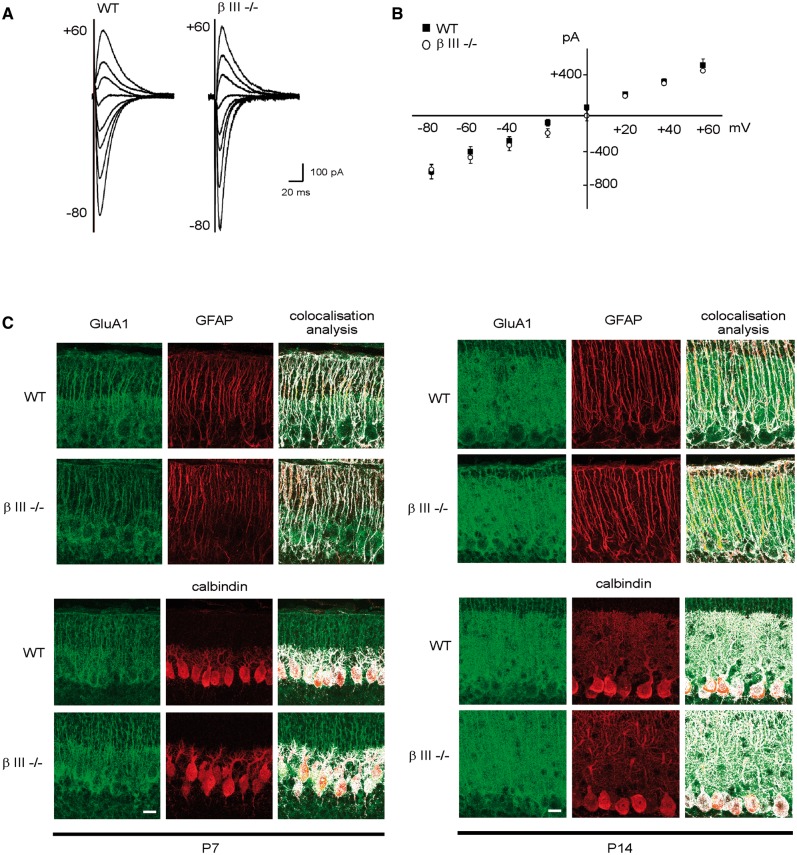
No change in GluA1-containing AMPA receptors in young βIII^-/-^ Purkinje cells. (**A)** Superimposed synaptic currents evoked at various holding potentials (-80 to +60 mV; 20 mV increments). (**B)** Current-voltage relationship for EPSCs recorded from Purkinje cells with spermine in patch pipette. All data are means ± SEM, *N =* 3, *n =* 7 (WT) and 4 (βIII^-/-^). (**C)** Midline sagittal cerebellar sections from animals at postnatal day 7 and 14, immunostained with anti-GluA1 antibody. Bar, 50 μm, *N =* 3 for both genotypes.

During normal development in the cortex and hippocampus the composition of AMPA receptor changes, with calcium permeable GluA1-containing receptors present early in development being replaced during maturation by calcium impermeable GluA2-containing receptors (22,23). Failure to switch from GluA1 to GluA2, or to edit GluA2 subunits would result in persistent expression of Ca^2+^-permeable AMPA receptors and EPSCs with larger amplitudes (24–27). Similarly, in the cerebellum it has been reported that by P30 GluA1 is no longer expressed in neurons and is exclusively expressed in Bergmann glia (28). Therefore, to determine whether an abnormal AMPA receptor composition is involved in the enhanced PF-PC EPSC amplitudes observed in Purkinje cells from β-III^-/-^ mice we carried out whole-cell voltage-clamp recordings from 3-week old animals. Inward rectification, a characteristic of receptors lacking GluA2, due to their voltage-dependent block by intracellular polyamines was not observed for either genotype at 3-weeks of age (Fig. 2A and B). In addition, equivalent abundance and cellular distribution of GluA1 immunoreactivity was observed in the molecular layer of β-III^-/-^ animals when compared to wild type animals (Pearson’s correlation coefficient (R ± SEM) for GluA1 colocalization: with GFAP P7, WT 0.78 ± 0.55, β-III^-/-^ 0.77 ± 0.54; P14, WT 0.75 ± 0.53, β-III^-/-^ 0.77 ± 0.55; with calbindin P7, WT 0.82 ± 0.58, β-III^-/-^ 0.81 ± 0.57; P14, WT 0.8 ± 0.46, β-III^-/-^ 0.81 ± 0.47; Fig. 2C). Together, these data reveal no difference in the expression profile of GluA1 in β-III^-/-^ animals, with normal edited GluA2-containing receptor compositions in mature β-III^-/-^ Purkinje cells.

### Loss of GLAST accentuates motor deficits in young β-III^-/-^ animals

Since a progressive loss of GLAST protein was found to correlate with worsening motor deficits and Purkinje cell loss in β-III^-/-^ animals (13) we investigated whether loss of GLAST is instrumental in disease progression. This was achieved by carrying out the first longitudinal behaviour analysis of GLAST knockout mice using gait analysis, rotarod and an elevated beam task.

**Figure 3.**
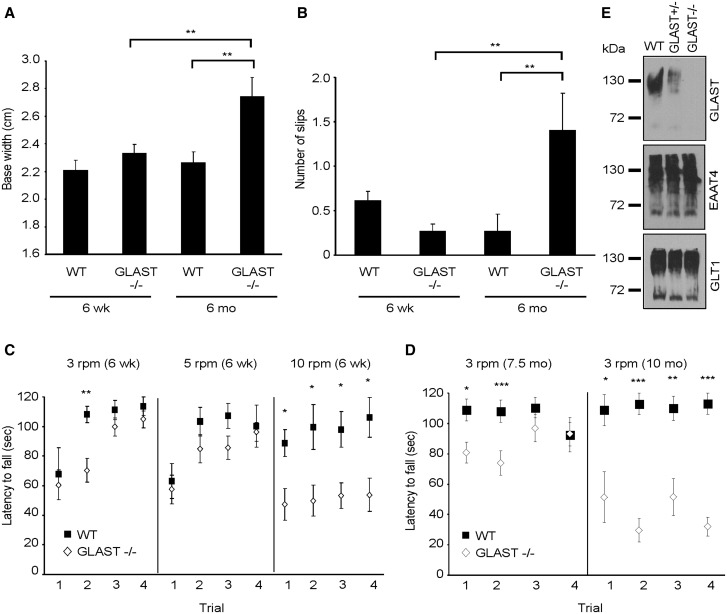
Progressive motor impairment in GLAST^-/-^ mice. (**A)** Hind limb base width of 6-week and 6-month old animals. (**B)** Number of hind-limb slips 6-week and 6-month old mice made when crossing narrow, elevated beam. **C,** Latency of 6-week old animals to fall from rotarod at 3-, 5- and 10-rpm. (**D)** Latency of 7.5- and 10-month old WT and GLAST^-/-^ animals to fall from rotarod at 3-rpm. All data are means ± SEM. *N =* 14 (WT), 14 (young GLAST^-/-^), 7 (old GLAST^-/-^) in all panels. **E,** Immunoblot analyses of cerebellar homogenates from 6-week old WT, GLAST^+/-^ and GLAST^-/-^ animals.

There was no difference in hind-limb base width (*P =* 0.618; Fig. 3A), number of slips off the elevated beam (*P =* 0.907; Fig. 3B) or ability to stay on rotarod at 3- and 5-rpm (Fig. 3C) in 6- week old GLAST^-/-^ animals when compared to age-matched WT animals. The main significant deficit observed in young GLAST^-/-^ animals, compared to age matched WT mice, is their ability to remain on the rotarod at 10-rpm (*P =* 0.035, 0.046, 0.039, 0.025, trial 1–4, respectively; Fig. 3C). However, it may be that young GLAST^-/-^ animals are slightly poorer at learning motor tasks than WT animals shown by a potential learning deficit at 3 rpm (Fig. 3C). By 6-months of age GLAST^-/-^ animals have a significantly wider hind-limb base width than when they were 6-weeks old (p= 0.01; Fig. 3A) and wider than 6-month old WT animals (*P =* 0.004). They make a greater number of slips on the elevated beam when 6-months old compared to age-matched WT (*P =* 0.003; Fig. 3B) and to when they were 6-weeks old (p= 0.002). Finally by 7.5-months of age they are worse on the rotarod at 3-rpm on day 1 and 2 of testing (*P =* 0.035, 0.008, respectively) and never attain WT performance level, on any day of testing at 10 months of age (*P =* 0.02, 0.0002, 0.005, 0.0002; Fig. 3D).

**Figure 4.**
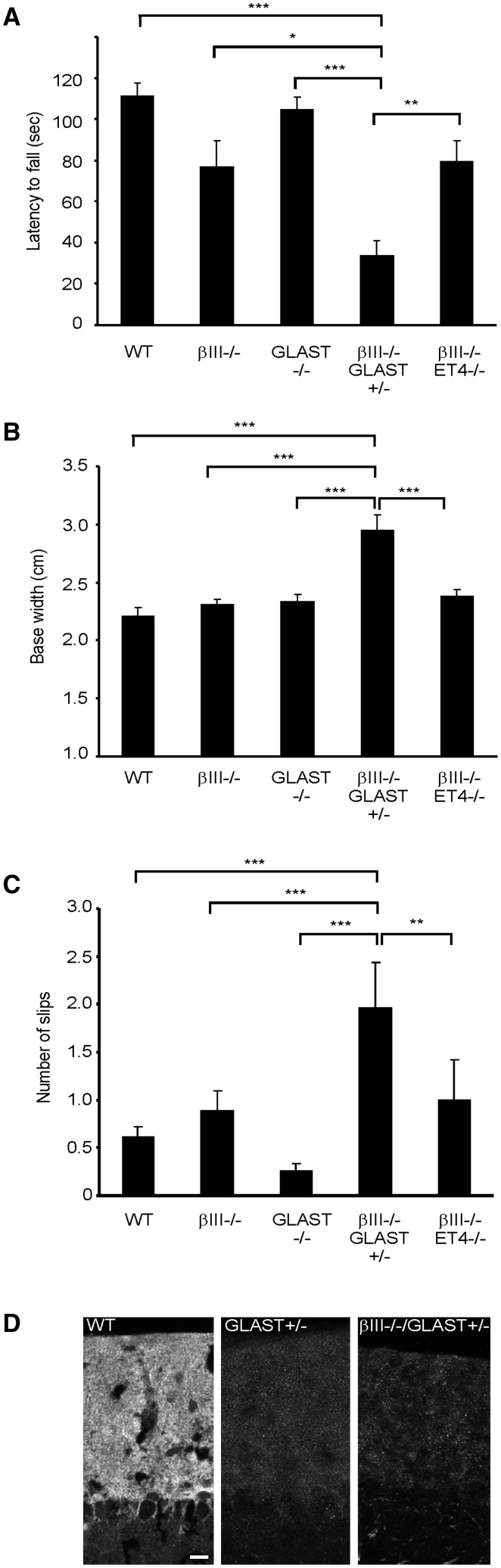
Motor decline of βIII^-/-^ animals accelerated by additional early loss of GLAST. (**A**) Latency of 6-week-old mice to fall from rotarod at 3-rpm. (**B**) Hind limb base width of 6-week-old animals. (**C**) Number of hind-limb slips 6-week old mice made when crossing narrow, elevated beam. All data are means ± SEM, *N =* 14 (WT), 7 (βIII^-/-^), 14 (GLAST^-/-^), 8 (βIII^-/-^/GLAST^+/-^), 8 (βIII^-/-^/ET4^-/-^). (**D**) Coronal sections of posterior cerebellum from 2-month-old mice immunostained with anti-GLAST antibody. Bar, 50 μm.

Moreover, analyses of young β-III^-/-^/GLAST^+/-^ animals, arising from crossing GLAST^-/-^ animals with β-III^-/-^ mice, revealed a worse performance on the rotarod at 3 –rpm compared to age-matched WT, β-III^-/-^, GLAST^-/-^ and β-III^-/-^/ET4^-/-^ animals (*P =* 7.73 × 10 ^−^ ^6^, 0.013, 8.55 × 10 ^−^ ^7^, 0.004; Fig. 4A). Similarly, young β-III^-/-^/GLAST^+/-^ animals had a significantly wider hindlimb base width (*P =* 7.6 × 10 ^−^ ^6^, 1.4 × 10 ^−^ ^5^, 2.5 × 10 ^−^ ^4^, 4 × 10 ^−^ ^4^; Fig. 4B) and made more slips on an elevated beam (*P =* 1.1 × 10 ^−^ ^5^, 3.8 × 10 ^−^ ^4^, 8.82 × 10 ^−^ ^7^, 0.01; Fig. 4C) than WT, β-III^-/-^, GLAST^-/-^ and β-III^-/-^/ET4^-/-^ mice, respectively. There was also a significant difference in motor phenotype of young β-III^-/-^/GLAST^+/-^ animals to both young EAAT4^+/-^ and GLAST^+/-^ animals (data not shown). Finally confocal immunofluorescence microscopy confirmed levels of GLAST protein in young β-III^-/-^/GLAST^+/-^ animals were similar to that of GLAST^+/-^ animals (Fig. 4D). Comparison of double mutant β-III^-/-^/GLAST^-/-^ animals was prevented due to non-Mendelian offspring genotypes arising from genetic crosses and therefore an insufficient number of animals with this genotype were obtained.

In contrast no difference was observed between the motor phenotype of young β-III^-/-^ and β-III^-/-^/ET4^-/-^ mice using rotarod (*P =* 0.999; Fig. 4A), hindlimb base width (*P =* 0.963; Fig. 4B) and elevated beam (*P =* 0.974; Fig. 4C) analyses. Moreover hindlimb base width measurements in 1-year old animals indicated a similar disease progression in β-III^-/-^/ET4^-/-^ mice (3.1 ± 0.17 cm) to that of β-III^-/-^ animals (3.12 ± 0.11 cm, *P =* 0.919) but an exacerbated phenotype in β-III^-/-^/GLAST^+/-^ animals (3.63 ± 0.13 cm, *P =* 0.005).

Together, this behavioural data clearly illustrates for the first time that loss of GLAST results in a progressive ataxic phenotype and it provides the first direct evidence that a reduction in GLAST protein levels acts in synergy with loss-of β-III spectrin function to accentuate motor decline, and is not simply an inconsequential side effect.

### Loss of GLAST accelerates Purkinje cell loss in young β-III^-/-^ animals

**Figure 5.**
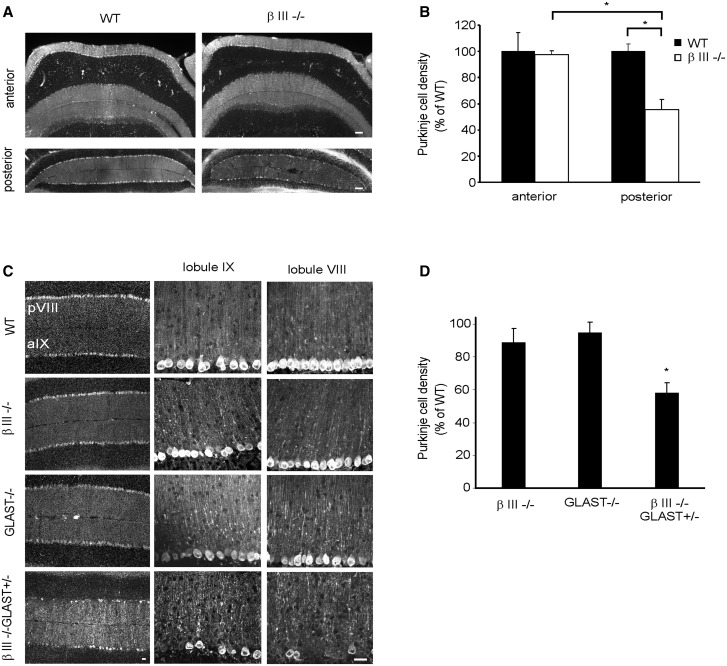
Purkinje cell loss in posterior lobules of βIII^-/-^ mice accelerated by additional early loss of GLAST. (**A)** Coronal cerebellar sections from 1-year-old WT and βIII^-/-^ mice immunostained with anti-calbindin antibody. (**B)** Quantification of Purkinje cell density in 1-year-old WT and βIII^-/-^ mice. (**C)** Representative confocal images, from coronal sections, of lobules VIII and IX from 3-month-old mice immunostained with anti-calbindin antibody. (**D)** Quantification of mean Purkinje cell density in lobules VIII, IX, X and Crus II of hemispheres. All data are means ± SEM, *N =* 3 for each genotype. Bar, 50 μm.

To determine whether loss of GLAST is also a key factor in the death of β-III^-/-^ Purkinje cells, we examined the Purkinje cell density in the posterior cerebellum of 3-month old β-III^-/-^/GLAST^+/-^ mice. The posterior cerebellum was chosen for this temporal analysis as even at 1-year of age in β-III^-/-^ mice we observe no significant loss of Purkinje cells within the anterior cerebellum compared to WT (*P =* 0.996; Fig. 5A and B) whereas significant cell death is evident in the posterior cerebellum when compared to WT (*P =* 0.029) and to the anterior cerebellum of β-III^-/-^ mice (*P =* 0.039). Quantification revealed there was cell loss in 3-month old β-III^-/-^/GLAST^+/-^ mice compared to age-matched WT animals (*P =* 0.035), whereas no significant loss was observed for either β-III^-/-^ or GLAST^-/-^ animals (*P =* 0.835, 0.970; Fig. 5C and D). These results again demonstrate a synergistic effect of reduced GLAST levels and loss-of β-III spectrin function resulting in diminished Purkinje cell survival. We see no Purkinje cell loss in either EAAT4^+/-^ or GLAST^+/-^ animals (data not shown).

### Loss of GLAST is not a downstream consequence of early EAAT4 loss

**Figure 6.**
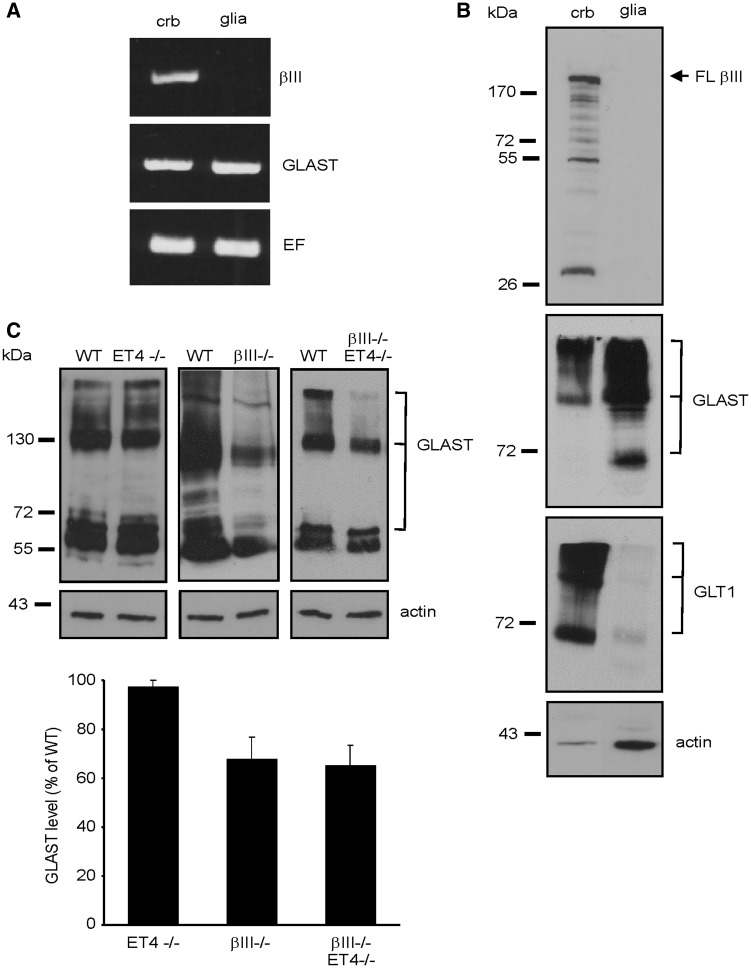
EAAT4 loss does not result in loss of GLAST. (**A**) Semi-quantitative RT-PCR analysis for βIII-spectrin and GLAST using RNA template extracted from cerebellar tissue (crb) or primary glial cultures (glia). Amplification of elongation factor (EF1A1) controlled for total template levels. (**B**) Immunoblot analysis of 10 µg of cerebellar and primary glial culture homogenate (arrow, full length (FL) βIII-spectrin, lower MW bands degradation products). (**C**) Top, Immunoblot analyses of cerebellar homogenate from 6-month old WT, ET4^-/-^, βIII^-/-^ and βIII^-/-^/ET4^-/-^ animals. Bottom, Densitometry data quantifying GLAST protein levels, normalised to actin and expressed as percentage of WT levels. *N =* 4 for each genotype. All data are means ± SEM.

The mechanism for loss of GLAST protein in β-III^-/-^ mice is not known but thought to be dependent on Purkinje cell dysfunction; immunofluorescence studies within the cerebellum have so far only shown β-III spectrin to be expressed in Purkinje cells (13,29). To ascertain whether there is any expression of β-III spectrin in Bergmann glia, and there is a cell-autonomous effect of β-III spectrin loss on GLAST protein levels, we carried out semi-quantitative RT-PCR and immunoblot analysis using extracts from primary cerebellar glial cultures and total cerebellar homogenates. No β-III spectrin transcript (Fig. 6A) or protein (Fig. 6B) was detected in primary cerebellar glial cultures, the purity of which was confirmed by the low level of GLT1 protein expression (Fig. 6B), also an astroglial glutamate transporter but the expression of which is highly-dependent on the co-culturing with neurons (30–32). These results indicate that β-III spectrin is unlikely to be expressed in Bergmann glia *in vivo* and demonstrate that in β-III^-/-^ animals a non-cell autonomous effect most likely underlies loss of GLAST in Bergmann glia.

To determine whether the later loss of GLAST is a downstream consequence of earlier EAAT4 loss from Purkinje cells, possibly mediated by excessive activation of Bergmann glial AMPA receptors (33), we examined GLAST levels in 6-month old ET4^-/-^ mice by immunoblot analyses. No loss was detected compared to WT animals (*P =* 0.38; Fig. 6C). Furthermore, when compared to β-III^-/-^ animals no additional loss of GLAST protein was observed in 6-month old β-III^-/-^/ET4^-/-^ mice (*P =* 0.84), corroborating the same rate of disease progression observed in β-III^-/-^ and β-III^-/-^/ET4^-/-^ animals (Fig. 4). Since the spectrin-based cytoskeleton has a multitude of functions mediated by interactions with various proteins (34,35) additional downstream consequences of loss-of β-III spectrin function in synaptic and structural integrity likely underpin the subsequent loss of GLAST in Bergmann glia.

### Dendritic degeneration in β-III^-/-^ animals is greater in posterior Purkinje cells with proximal dendrites being the most susceptible

**Figure 7.**
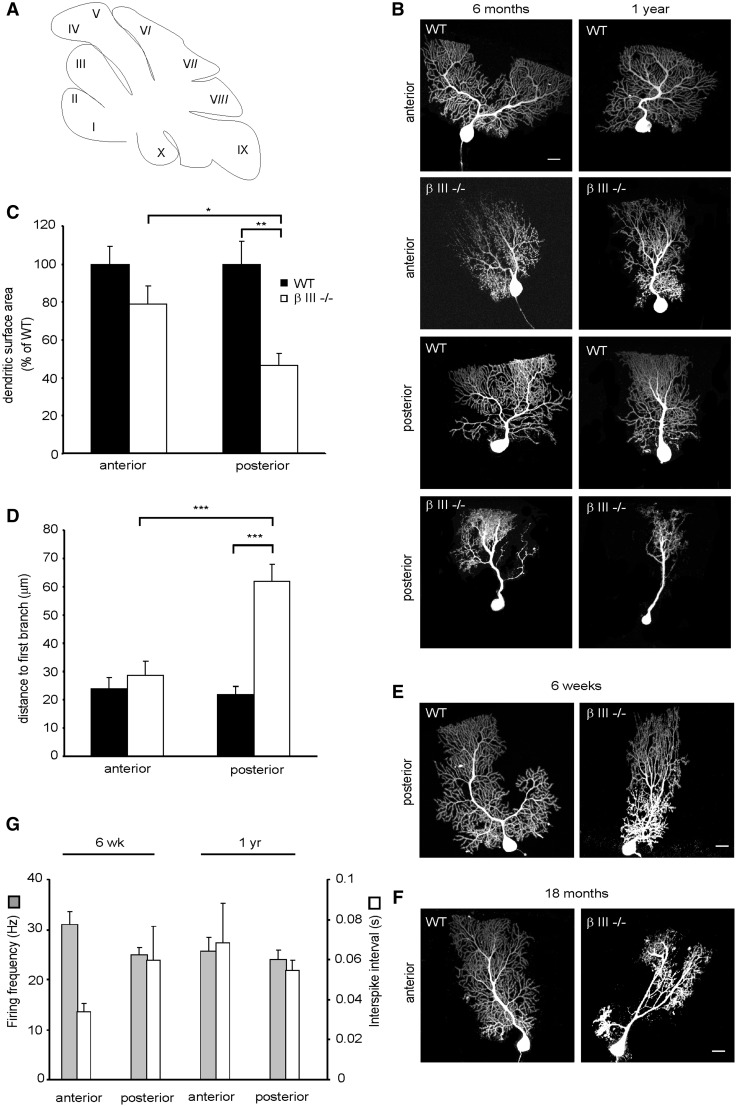
Proximal dendrites of posterior βIII^-/-^ Purkinje cells most vulnerable to dendritic degeneration. (**A**) Schematic of sagittal section of cerebellar lobules. (**B**) Representative confocal images of Purkinje cells filled with Alexa Fluor 568 from anterior (II –V) and posterior (VIII-X) lobules of 6-month and 1-year-old WT and βIII^-/-^ animals. (**C**) Quantification of dendritic surface area of individual Purkinje cells from 6-month old WT and βIII^-/-^ mice. *N =* 3, *n =* 4 (WT), 6 (βIII^-/-^) for each region. (**D**) Quantification of distance from Purkinje cell soma to first dendritic branch point in animals > 6-months of age. *N =* 9, *n =* 14 (WT), 18 (βIII^-/-^). (**E**) Representative confocal images of Purkinje cells filled with Alexa Fluor 568 from posterior (VIII-X) lobules of 6-week old WT and βIII^-/-^ animals. (**F**) Representative confocal images of Purkinje cells filled with Alexa Fluor 568 from anterior lobules (II –V) of 18-month- old WT and βIII^-/-^ animals. (**G**) Spontaneous firing frequency and interspike interval of Purkinje cells from young [N= 4, *n =* 12 (anterior), 19 (posterior)] and old βIII^-/-^ animals [*N =* 9, *n =*28 (anterior), 30 (posterior)]. All data are means ± SEM. Bar, 20 μm.

Finally to investigate further the regional difference in Purkinje cell death in β-III^-/-^ mice (Fig. 5) we filled, by diffusion from a whole-cell patch pipette, individual Purkinje cells in acute cerebellar sagittal slices with a fluorescent dye. Cells were visualized by confocal microscopy and the dendritic surface area of individual Purkinje cells measured from the anterior (lobules II, III, IV, V) and posterior (VIII, IX, X) lobules (Fig. 7A). This revealed in 6-month old animals a significant difference in the extent of dendritic degeneration between β-III^-/-^ Purkinje cells from the posterior and anterior lobules (*P =* 0.05) and between posterior β-III^-/-^ and WT cells (*P =* 0.006) but not between anterior β-III^-/-^ and WT cells (*P =* 0.248; Fig. 7B and C). However, by 1-year of age, there is a significant difference in the dendritic surface area between β-III^-/-^ and WT cells from anterior lobules (60.9 ± 4.9% of WT, *P =* 0.025, *N =* 5, *n =* 7 (WT), 9 (β-III^-/-^) and further degeneration of β-III^-/-^ cells from posterior lobules [35.6 ± 6% of WT, *P =* 1.5 × 10 ^−^ ^5^, *N =* 5, *n =* 12 (WT), 9 (β-III^-/-^)].

**Figure 8.**
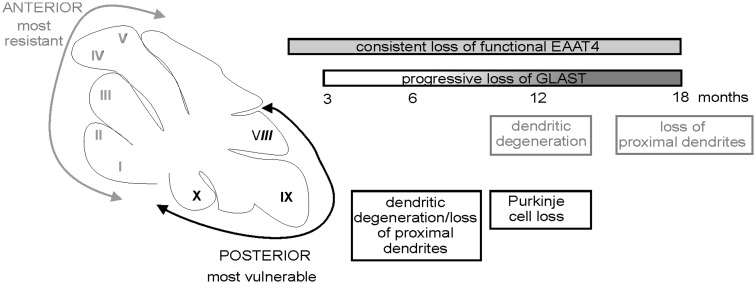
Differences in the timeline of degeneration between anterior and posterior cerebellar lobules in a mouse model of SCA5/SPARCA1.

Images of individually filled Purkinje cells were also used to measure the distance from the cell body to the first branch point and this revealed that in animals > 6-months of age this distance was significantly larger in β-III^-/-^ cells from posterior lobules compared to WT cells (*P =* 4.0 × 10 ^−^ ^5^) and β-III^-/-^ cells in anterior lobules (*P =* 2.4 × 10 ^−^ ^7^). However, there was no difference in distance from the cell body to the first branch point between WT and β-III^-/-^ cells in anterior lobules (*P =* 0.988; Fig. 7B and D). The absence of proximal dendrites in β-III^-/-^ Purkinje cells from posterior lobules was not a developmental defect as they were present in 6-week old β-III^-/-^ mice (Fig. 7E). Moreover, some Purkinje cells (3 out of 9 cells) in anterior lobules exhibited some loss of proximal dendrites at 18-months of age highlighting a progression of proximal dendritic degeneration (Fig. 7F). Together the data reveal that proximal dendrites are the most susceptible to degeneration, starting within the posterior cerebellum but progressing to the anterior cerebellum, identifying a novel feature of disease pathology (Fig. 8).

Finally, we previously reported that in acute slices the spontaneous activity of β-III^-/-^ Purkinje cells were about half that of WT cells, both at 6-weeks and 6-months of age (13). However, within this study no distinction was made between posterior and anterior lobules. In light of our current observation that the degeneration of the posterior β-III^-/-^ Purkinje cells is earlier and greater than that of the anterior Purkinje cells we sought to determine whether there was any variation in the reduction of spontaneous activity in the different populations of β-III^-/-^ Purkinje cells. No significant difference in frequency was observed within the different populations in young or old β-III^-/-^ animals (Fig. 7G) and a similar regularity of firing was observed in all populations (coefficient of variation < 0.2). Nevertheless, it was cells in anterior lobules from 6-week old β-III^-/-^ mice, those exhibiting the least amount of degeneration, that were found to have the highest spontaneous activity and therefore the closest to that of age-matched WT cells (41.7 ± 4.6 Hz, 0.02 ± 0.003 s).

## Discussion

This study conclusively demonstrates that loss of EAAT4 underpins early Purkinje cell hyper-excitability in β-III^-/-^ animals and is the first study to directly show that loss of GLAST accentuates motor deficits and Purkinje cell death, appearing to act in synergy with loss-of β-III spectrin function. Moreover the posterior cerebellum appears to be especially vulnerable to the synergistic effect, likely involving the combined loss of functional EAAT4 and GLAST (Fig. 8). Yet, the observed loss of GLAST in β-III^-/-^ animals does not appear to arise from the early loss of EAAT4 protein.

### Loss of β-III spectrin leads to complete loss of functional EAAT4 resulting in Purkinje cell hyper-excitability

Several studies, including our own, have shown EAAT4 levels to be reduced early in various mouse models of ataxia (13,36–38). For example, we observed loss of EAAT4 in β-III^-/-^ mice, a mouse model of SCA5/SPARCA1, before any Purkinje cell degeneration (13). Similarly reduced EAAT4 levels were observed in young Spnb3^-/-^ mice, another mouse model of SCA5, with EAAT4 accumulating in the cell soma and dendritic shafts (38), a feature reported in SCA5 autopsy tissue (11). Loss of EAAT4 was also found in a mouse model of spinocerebellar ataxia type I (SCA1) prior to any sign of disease (36,39) and EAAT4 levels were found to be decreased in a spontaneous mouse model (*staggerer*) of ataxia (37). These correlative findings have long suggested that EAAT4 loss may play a key role in ataxia but direct evidence corroborating this association has been lacking.

Here, using EAAT4 knockout mice, we have shown that the greater excitation we observe in young β-III^-/-^ mice are due to loss of EAAT4. A similar increase in peak amplitude of PFPC-EPSCs was observed in young ET4^-/-^ mice as seen in β-III^-/-^ mice (13) and moreover there was no additive effect in β-III^-/-^/ET4^-/-^ mice when compared to ET4^-/-^ and β-III^-/-^ mice. Together, these data provide compelling evidence that the greater excitation arising from loss-of β-III spectrin function is due to loss of EAAT4 and reduced post-synaptic glutamate uptake. Moreover, despite β-III^-/-^ mice still expressing about 50% of WT EAAT4 protein levels the increase in Purkinje cell excitability is the same for ET4^-/-^ and β-III^-/-^ animals. There is also no difference in motor phenotype between β-III^-/-^ and β-III^-/-^/ET4^-/-^ mice. It would therefore appear that in the absence of β-III spectrin the remaining EAAT4 protein is not functional. This is an important finding as it suggests that even if levels of EAAT4 could be elevated pharmacologically or through genetic re-introduction it would be unlikely to lead to any therapeutic benefit as without the β-III spectrin anchor the EAAT4 protein would not be correctly targeted or maintained at the plasma membrane.

Our observed change in peak amplitude of PFPC-EPSCs in ET4^-/-^ mice is in contrast to two earlier studies (40,41). However, the animals used in each study were of different ages; Nikkuni et al using 18–22 day old mice (41), Takayusu et al 22-42 day old (40), while the present study used 42–46 day old mice. Furthermore, differences in recording conditions (temperature and holding potential) between the three studies also hinder direct comparison and could account for variations in observations. In addition the varying molecular composition of Purkinje cells from different sagittal compartments (42–44) has the possibility of further compounding variability between different data sets.

### Loss of β-III spectrin and GLAST act in synergy to exacerbate motor decline

Similar to EAAT4, correlative findings have also suggested loss of GLAST may play a role in the progression of ataxia, but again direct evidence has been lacking. Correlative findings include loss of GLAST and reduced astroglial glutamate uptake when mutant ataxin-7 is solely expressed in Bergmann glia with this mouse displaying ataxia and Purkinje cell death (45). Further correlative evidence comes from conditional ablation of Bergmann glia in adult mice resulting in ataxia and degeneration of Purkinje cell dendrites (46) and loss of GLAST observed in mouse models of SCA1 (47) and SCA5 (13) at later stages of disease.

We have carried out the first longitudinal study using GLAST knockout mice and have shown that loss of GLAST gives rise to progressive ataxia. Previously it had been reported that GLAST deficient mice display mild motor deficits, but the age of mice was not reported (19). Here we show a mild motor defect in young (6-weeks of age) GLAST^-/-^ animals but reveal that the motor deficits are progressive, with old animals performing much worse on all motor tasks. Moreover, through genetic crosses we directly show for the first time that reducing GLAST levels has a severe consequence on the severity of ataxia in β-III^-/-^ animals with young β-III^-/-^/GLAST^+/-^ mice displaying a much earlier decline in motor performance than either β-III^-/-^ or GLAST^-/-^ animals highlighting a synergistic effect of reduced GLAST and loss-of β-III spectrin function, likely due to a combined loss of EAAT4 and GLAST, the two predominant glutamate transporters in the cerebellum. This conclusion is supported by our analysis of young EAAT4^-/-^/GLAST^-/-^ double knockouts that show the same level of motor dysfunction as β-III^-/-^/GLAST^+/-^ mice (data not shown).

### Survival of β-III^-/-^ Purkinje cells in posterior cerebellum dependent on high levels of both EAAT4 and GLAST

The fact loss of Purkinje cells was observed within the posterior cerebellum of young β-III^-/-^/GLAST^+/-^ and old β-III^-/-^ animals indicates that these cells are particularly vulnerable to the synergistic effect of loss of GLAST and β-III spectrin function. GLAST is believed to be present in functional excess throughout the cerebellum (48). In contrast EAAT4 displays a differential pattern of expression within parasagittal bands with Purkinje cells in posterior regions possessing higher levels of EAAT4 compared to the anterior regions of the cerebellum (42,44,49). It is therefore Purkinje cells that should normally express high levels of EAAT4 that appear to be more vulnerable to the combined loss of GLAST and β-III spectrin (hence EAAT4). Of note greater glutamate release was shown to occur from climbing fibre terminals in zebrin-positive regions, zones of high EAAT4 expressing Purkinje cells (50). Purkinje cell proximal dendrites, the site of climbing fibre innervation, in the posterior cerebellum may therefore be the most susceptible to degeneration following the combined loss of the two predominant glutamate transporters, EAAT4 and GLAST due to higher levels of glutamate exposure. It is yet to be determined if differences in the functional circuitry between zebrin-positive and negative bands underlie the selective progressive Purkinje cell susceptibility.

There is no significant difference in the reduction of spontaneous firing rate between β-III^-/-^ Purkinje cells with varying extents of cell degeneration. This finding indicates that there is no effect of cell atrophy on reduced voltage-gated sodium channel density following the loss of β-III spectrin function (13,51), in contrast to a reported compensatory effect of neuronal atrophy in a mouse model of SCA1 which restored BK channel density and intrinsic membrane excitability (52).

The molecular mechanism leading to the later but progressive loss of GLAST in β-III^-/-^ mice is unclear. Identifying this mechanism will be paramount in the development of an effective therapy since data from this study directly highlights the critical role loss of GLAST plays in disease progression.

### Relevance of posterior cerebellar pathology

The discovery that the posterior cerebellum is the first area affected pathologically in β-III^-/-^ mice provides additional insight into disease pathogenesis and possibly the specific cognitive deficits observed following complete loss of β-III spectrin function (12). Although there are anatomical differences between mice and humans with respect to cerebellar input and involvement of cerebellum in non-motor functions (53), if the human posterior cerebellum is similarly more vulnerable to loss of both EAAT4 and GLAST the functional connectivity between the prefrontal cortex and posterior cerebellum (54,55) would be disrupted in SPARCA1 patients. Of note the posterior cerebellar-prefrontal circuit is involved in cognitive tasks such as attention shifting and verbal working memory tasks (56,57) which appear to be especially impaired in patients with ataxia (58).

In addition the prefrontal cortex and the posterior cerebellum are believed to be significantly affected by ageing (59–61). The discovery that Purkinje cells within the posterior cerebellum are more sensitive to reduced levels of both EAAT4 and GLAST protein may also provide mechanistic insight into age-related decline in motor and cognitive ability. Developing therapeutic strategies to target the posterior cerebellum is likely to prove useful not only in alleviating motor and cognitive deficits associated with inherited ataxias but may also mitigate the effects of normal ageing.

## Materials and Methods

### Animals

All procedures involved in the generation and analysis of mutant mice were carried out according to the United Kingdom Animals (Scientific Procedures) Act (1986) and other Home Office regulations under specific-pathogen-free conditions. GLAST^-/-^, ET4^-/-^ and β-III^-/-^ mice, all on a C57BL/6 genetic background, were generated as described previously (13,19,43) and both sexes were used in all experiments. The genotypes of all experimental animals were confirmed by PCR analysis on genomic DNA extracted from ear notch biopsies using ChargeSwitch gDNA tissue kit (Invitrogen, Carlsbad, CA) as described previously (13) or as follows: for ET4^-/-^ mice a common upstream primer (5′-ttcctgattgctggaaagattctgg-3′) and primers specific for the wild-type allele (5′-agttcagggaaaggccataccttgg-3′) and the *PGK-neo* cassette in the mutant allele (5′-ggatcggccattgaacaagatgg-3′) were used for amplification. The 220-bp (from wild-type allele) and 1200-bp (from targeted allele) PCR products were resolved by electrophoresis on a 1.6% w/v agarose gel. For GLAST^-/-^ mice specific primer sets were used for amplification of wild-type allele (5′-aagtgcctatccagtccaacga-3′; 5′-aagaactctctcagcgcttgcc-3′) and mutant allele (5′-aatggaaggattggagctacgg-3′; 5′-ttccagttgaaggctcctgtgg-3′). The 214-bp (from wild-type allele) and 362-bp (from targeted allele) PCR products were resolved by electrophoresis on a 1.6% w/v agarose gel. All knockout mice were viable, although pups from GLAST^-/-^ mice were routinely fostered with CD1 mothers to ensure survival.

### Slice electrophysiology

PF-EPSC measurements at a range of stimuli (3-18 V, 200 μs duration) were recorded at room temperature as previously described (13) and the amplitudes and decay time constants (*tau*) of PF-evoked EPSCs measured using the NeuroMatic analysis program in IGOR Pro (Wavemetrics, Lake Oswergo, OR). Spontaneous action potentials from acute slices were recorded at 30˚C ± 2˚C as previously described (13). Sagittal cerebellar slices (200 µm) from P16-21 WT and β-III^-/-^ mice were used to determine I/V relationship of PF-EPSCs. Internal solution contained (in mM): 108 Cs Methanesulfonate, 9 NaCl, 9 HEPES, 1.8 EGTA, 1.8 MgCl_2_, 0.4 NaGTP, 2 MgATP, 63 sucrose and 5 QX-314, adjusted to pH 7.4 with CsOH. Recordings were made at room temperature and picrotoxin (50 μM) was added to the ACSF. Stimulus intensity was set to evoke PF-EPSCs of approximately 500 pA at V_h_ -60 mV (7-42 V, 200 μs). Spermine (0.1 mM) was included in the internal solution to replace dialysed endogenous polyamines. I/V relationships for PF-EPSCs in WT and β-III^-/-^ mice were generated by averaging 3 EPSCs at each holding potential (-80 mV to +60 mV in 20 mV increments). Series resistances were <15 MΩ and were compensated for by 85%. Data was acquired using pClamp 9 (Molecular Devices, Sunnyvale, CA) and recordings were filtered at 2 kHz and sampled at 10 kHz. Data analysis was carried out in Neuromatic, IGOR Pro (Wavemetrics, Lake Oswego, OR) and using in-house MatLab scripts.

### Motor coordination tests

Elevated beam, footprint analysis and rotarod were performed as previously described (13).

### Immunohistochemistry

Brains were removed and immersion-fixed with 4% w/v paraformaldehyde in 0.1 M sodium phosphate buffer, pH 7.4 overnight at 4 °C and either embedded in paraffin or cryoprotected by immersion in 0.1 M sodium phosphate buffer (pH 7.4) containing 30% w/v sucrose. Paraffin sections (10 μm-thick), mounted on poly-L-lysine-coated slides, were immunostained with anti-GluA1 antibody (Abcam). All other tissues were quick-frozen on dry-ice, then 30 μm-thick coronal free-floating cerebellar sections immunostained with either anti-calbindin (Swant) or anti-GLAST antibody as described previously (62). All quantification, carried out blind to genotype, involved counting the number of Purkinje cells in the anterior (lobules I-V, simplex and crus I) and posterior cerebellum (lobules VI -IX, crus II and flocculonodular lobe, lobule X) from three sections/animal and the counts averaged. Data were pooled from GLAST^-/-^ and β-III^+/-^/GLAST^-/-^ animals as no phenotype observed in β-III^+/-^ animals (62). Images were captured with either a Zeiss inverted LSM510 or Nikon confocal laser scanning microscope and co-localization analysis carried out using Image J.

### Cerebellar astrocyte culture

Primary astrocytes were purified from 6-day old WT mouse cerebella. The cerebella were dissected and incubated in papain for 30 min at 37 °C. After trituration in astrocyte media (DMEM high glucose, 2 mM glutamine, 10% v/v FBS) containing 0.02% w/v DNase cells were preplated for 20 min on an uncoated dish to remove contaminant fibroblasts. Unattached cells were transferred to a dish coated with 0.5 µg/ml of poly-D-Lysine for 1 h to allow astroglial attachment. Cells were rinsed with PBS to remove neurons and were cultured in DMEM with 10% v/v FBS. Cultures were passaged after 2-3 DIV.

### Semiquantitative RT-PCR

Total RNA was extracted from mouse cerebellum or primary glial cultures using RNeasy Mini kit (Qiagen, Valencia, CA) and RT-PCRs carried out using One-Step RT-PCR kit (Qiagen) according to manufacturer’s instructions. Primers for RT-PCR reactions were: β-III spectrin F1 5′-atgagcagcactctgtcacccact-3′, R7 5′-gccaattcttttgcctt ccacagc-3′; GLAST For 5′-atgacaaaaagcaacggagaag-3′, Rev 5′-ctacatcttggtttcgctgtc-3′. Amplification of the ubi quitously expressed elongation factor alpha was used to control for RNA levels (Stratagene, La Jolla, CA).

### Immunoblotting

Protein extracts were prepared from whole cerebella or primary glial cultures and processed as previously described (13). Primary antibodies were goat anti-βIII spectrin (1:1000; Santa Cruz), rabbit anti-GLAST (1:200), -GLT1 (1:4,000) or mouse anti-actin, -calbindin (1:1,600; Sigma, St. Louis, MO). Secondary antibodies were HRP-conjugated donkey anti-rabbit IgG, HRP-conjugated sheep anti-mouse IgG (1:4,000; Amersham Pharmacia) and HRP-conjugated donkey anti-goat IgG (1:4,000; Santa Cruz).

### Purkinje cell filling

Individual Purkinje cells were filled with 0.02 mM Alexa FluorAR 568 hydrazide (Invitrogen, A-10441) and imaged as described previously (29). The surface area was quantified using in-house MatLab scripts and distance to first branch point measured on z-projections in Image J.

### Statistics

EPSC data were analysed using a mixed model ANOVA with post-hoc Tukey’s HSD for between subjects comparisons. All other data were assessed either by Kruskal-Wallis or ANOVA followed by Bonferroni-corrected Mann-Whitney and Tukey’s HSD post-hoc tests, respectively. Significance was accepted at *P*-values of 0.05 or less. *N =* number of animals and *n =* number of cells.
